# Discriminatory profile of rDNA sites and trend for acrocentric chromosome formation in the genus *Trachinotus* Lacépède, 1801 (Perciformes, Carangidae)

**DOI:** 10.3897/CompCytogen.v6i4.3062

**Published:** 2012-10-31

**Authors:** Uedson Pereira Jacobina, Marcelo Ricardo Vicari, Luiz Antonio Carlos Bertollo, Wagner Franco Molina

**Affiliations:** 1 Department of Cell Biology and Genetics, Centro de Biociências, Universidade Federal do Rio Grande do Norte, Campus Universitário, 59078 – 970, Natal, RN, Brazi; 2 Department of Structural, Molecular Biology and Genetics, Universidade Estadual de Ponta Grossa, Ponta Grossa, PR, Brazil; 3 Department of Genetics and Evolution, Universidade Federal de São Carlos, Via Washington Luiz, Km 235, 13565 – 905, São Carlos, São Paulo, Brazil

**Keywords:** Carangidae, 18S rDNA, 5S rDNA, cytotaxonomic markers, evolutionary pathways

## Abstract

Chromosomal traits have provided valuable information for phylogeny and taxonomy of several fish groups. Three Atlantic Carangidae species of the genus *Trachinotus* Lacépède, 1801 (*Trachinotus goodei* Jordan et Evermann, 1896, *Trachinotus carolinus* (Linnaeus, 1766)and *Trachinotus falcatus* (Linnaeus, 1758)) were investigated, having 2n=48 chromosomes but different chromosomal arms (FN number), i.e., 52, 56 and 58, respectively, in view of the different number of two-armed chromosomes found in their karyotypes. Thus, *Trachinotus goodei*, *Trachinotus carolinus* and *Trachinotus falcatus* present a progressive distancefrom the probable basal karyotype proposed for Perciformes (2n=48 acrocentrics, FN=48). At first sight, these findings do not agree with the phylogenetic hypothesis based on mitochondrial sequences, where *Trachinotus goodei* appear as the most derived species, followed by *Trachinotus falcatus* and *Trachinotus carolinus*, respectively. However, the chromosomal mapping of ribosomal DNAs was informative for clarifying this apparent conflict. Indeed, the multiple 5S and 18S rDNA sites found in *Trachinotus goodei* corroborate the most derived condition for this species. In this sense, the occurrence of the unexpected number of two-armed chromosomes and FN value for this species, as well as for *Trachinotus carolinus*, must be due to additional rounds of acrocentric formation in these species, modifying the macrostructure of their karyotypes.

## Introduction

The genus *Trachinotus* Lacépède, 1801, also known as pompanos, encompasses 20 species distributed in tropical and subtropical oceans (Cunha 1981). In the Eastern Atlantic, the species *Trachinotus carolinus* (Linnaeus, 1766), popular for both sport and commercial fishing, *Trachinotus falcatus* (Linnaeus, 1758), a game fish species, and *Trachinotus goodei* Jordan et Evermann, 1896, a species with a high potential for aquaculture and sport fishing, are the most widely distributed, occurring from the Southern United States to Northern Argentina (McMaster 1988, Lazo et al. 1998, Heilman and Spieler 1999). Recent data identified population differentiations in the number and positions of the ribosomal sites among the extensively distributed species, *Trachinotus falcatus* and *Trachinotus goodei* (Accioly et al. in press). Indeed, there is growing evidence that cytotaxonomic markers, particularly ribosomal sites, may reveal the genetic structure of marine fish populations (Motta-Neto et al. 2011a, Lima-Filho et al. in press).

In addition to their biological significance in commercial and sport fishing, representatives of the genus *Trachinotus* are considered potentially suitable for pisciculture purposes (Watanabe 1995, Weirich et al. 2006). *Trachinotus* species have very desirable biological characteristics, such as fast adaptation to confined environments, good tolerance to extreme environmental conditions and rapid growth (Jory et al. 1985). Nevertheless, genetic and cytogenetic foundations supporting their cultivation remain largely unknown.

Most species of the marine Perciformes exhibit a basal karyotype composed of 2n=48 acrocentric chromosomes, extensively conserved in several families (Molina 2007). Given the large number of species, most cytogenetic studies have focused on mapping biodiversity in this order, the largest of all living vertebrates. Among the family Carangidae, cytogenetic data have already been reported for a total of 27 species in 13 genera (e.g. Caputo et al. 1996, Sola et al. 1997, Rodrigues et al. 2007, Chai et al. 2009). Of these, few species occur exclusively in the Atlantic. The present cytogenetic study characterizes the species *Trachinotus* c*arolinus*, *Trachinotus falcatus* and *Trachinotus goodei* through conventional staining, Ag-NOR detection, C-banding, CMA_3_/DAPI fluorochrome staining, and mapping of the 18S and 5S rDNA sequences by dual-color FISH. Useful phylogenetic information was provided by ribosomal sequences mapping, indicating an intriguing scenario with additional acrocentrics formation in *Trachinotus goodei* and *Trachinotus carolinus*.

## Material and methods

Samples of the species *Trachinotus carolinus* (N=5; 3 males. one female, one immature), *Trachinotus falcatus* (N=10; 4 males, 3 females, 3 immatures) and *Trachinotus goodei* (N=10; 6 males, 4 females) were obtained on the coast of Rio Grande do Norte state (05°05'26"S, 36°16'31"W), in Northeast Brazil. Prior to chromosomal preparations, specimens were submitted to *in vivo* mitotic stimulation for 24 hours, through intramuscular and intraperitoneal injection of complex antigens (Molina et al. 2010). Individuals were anesthetized with clove oil (Griffiths 2000) and sacrificed. Mitotic chromosomes were acquired from cell suspensions of anterior kidney fragments according to *in vitro* mitotic block (Gold et al. 1990). Cell suspensions were dripped onto slides coated with a film of distilled water heated to 60°C, and stained with 5% Giemsa diluted in a phosphate buffer pH 6.8. The material was analyzed under 1000× magnification and the best metaphases were photographed under an Olympus BX50® epifluorescence microscope, with an Olympus DP70® digital image capturing system. About 30 metaphases were analyzed for each individual in order to determine the diploid number for every species.

### Chromosome nomenclature

Chromosomes were classified as metacentric (m), submetacentric (sm), subtelocentric (st) and acrocentric (a), based on the system proposed by Levan et al. (1964).

### Chromosome banding

The heterochromatic and nucleolar organizer regions (Ag-NORs) were identified using techniques developed by Sumner (1972) and Howell and Black (1980) respectively. CMA_3_/DAPI staining was applied in accordance with Barros-e-Silva and Guerra (2010).

### Cytogenetic mapping protocols

Two probes were used: an 18S rDNA probe obtained from the nuclear DNA of *Prochilodus argenteus* Spix et Agassiz, 1829 (Hatanaka and Galetti 2004); a 5S rDNA probe isolated from the genomic DNA of *Leporinus elongatus* Valenciennes, 1850 (Martins and Galetti 1999); probes were labeled by polymerase chain reaction (PCR), using biotin-16-dUTP (Roche Applied Science®) for 18S rDNA or digoxigenin-11-dUTP (Roche Applied Science®) for 5S rDNA. PCR labeling for rDNA clones was performed with specific primers, using 20 ng of template DNA, 1X *Taq* reaction buffer (200 mM Tris pH 8.4, 500 mM KCl), 40 µM dATP, dGTP and dCTP, 28 µM of dTTP, 12 µM biotin-16-dUTP or digoxigenin-11-dUTP, 1 µM primers, 2 mM MgCl_2_ and 2 U of *Taq* DNA Polymerase (Invitrogen®) under the following conditions: 5 min at 94°C; 35 cycles: 1 min at 90°C, 1 min 30 s at 52°C and 1 min 30 s at 72°C; and a final extension step at 72°C for 5 min.

The overall hybridization procedure followed the protocol described by Pinkel et al. (1986), under high stringency conditions (2.5 ng/µL from each probe, 50% deionized formamide, 10% dextran sulphate, 2XSSC, pH 7.0 – 7.2, at 37°C overnight). After hybridization, slides were rinsed in 15% formamide/0.2XSSC at 42°C for 20 min, 0.1XSSC at 60°C for 15 min, and 4XSSC/0.05% Tween at room temperature for 10 min (two times for 5 min each). Signal detection was performed using streptavidin-alexa fluor 488 (Molecular Probes®) for the 18S rDNA probe; and anti-digoxigenin-rhodamine (Roche Applied Science®) for 5S rDNA, which were detected by dual color FISH.

## Results

All species analyzed exhibited 2n=48 chromosomes, however with a notable difference in the number of two-armed (bibrachial) elements.

**Figure 1.**
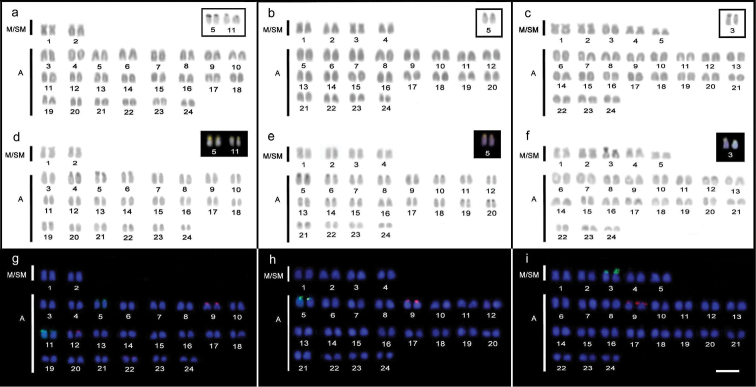
Karyotypes of *Trachinotus goodei* (**a, d, g**), *Trachinotus carolinus* (**b, e, h**) and *Trachinotus falcatus* (**c, f, i**). Conventional staining (**a, b, c**) highlighting the chromosomal pairs carrying Ag-NOR sites; C-banding (**d, e, f**); nucleolar organizer pairs are highlighted by staining with CMA_3_^+^/DAPI^-^. Dual-colorFISH (**f,  g,  h**) showing the chromosomal mapping of the 18S rDNA (green) and 5S rDNA (red) sites. Bar = 5 µm.

The karyotype of *Trachinotus goodei* (Figure 1a, d, g) is composed of 4 m/sm and 44a (FN=52). The heterochromatic regions in this species are very reduced and restricted to small blocks in the chromosomal pericentromeric regions. The Ag-NORs/18S rDNA sites were identified near the centromeric region of two acrocentric pairs, tentatively No. 5 and 11 of the karyotype. These sites proved to be rich in GC base composition (CMA^+^/DAPI^-^) (Figure 1d). Hybridization signals with 5S rDNA probes were also identified on the terminal regions of the short arms of three acrocentric pairs, tentatively numbered as 9, 12 and 22 (Figure 1g).

The *Trachinotus carolinus* karyotype (Figures 1 b, e, h) consists of 8m/sm and 40a (FN=56). The content of heterochromatin is also poorly distributed in the pericentromeric regions of some chromosome pairs. Ag-NORs/18S rDNA sites were located on the short arm of only one acrocentric pair, identified as number 5. These sites are clearly heterochromatic, with a CMA^+^/DAPI^-^ pattern. The 5S rDNA sites were mapped only on the short arm of the acrocentric chromosome 9.

The karyotype of *Trachinotus falcatus* (Figure 1c, f, i) has the largest number of bibrachial elements if compared to the other species, i.e., 10 m/sm and 38a (FN=58). As in the two previous species, small heterochromatic blocks are present in pericentromeric regions of the chromosomes. Ag-NORs/18S rDNA sites were situated in the terminal region of the short arm of the submetacentric chromosome pair 3, which also appears heterochromatic after C-banding, with a CMA^+^/DAPI^-^ pattern. The 5S rDNA sites were mapped exclusively on the short arms of the acrocentric pair 9.

## Discussion

As in many species of Perciformes, the species analyzed displayed 2n=48 and large numbers of acrocentric chromosomes, although there were notable differences in karyotype macrostructure. This is particularly evident for the number of chromosome arms (FN) that varies between species. Thus, *Trachinotus goodei* exhibits FN=52, *Trachinotus carolinus* FN=56 and *Trachinotus falcatus* FN=58. Karyotypes similar to those presented here for *Trachinotus goodei* and *Trachinotus falcatus* were previously identified in other populations of this species on the Southeast and Northeast coasts of Brazil (Rodrigues et al. 2007, Accioly et al. in press).

Evolutionary karyotype modifications resulting from pericentric inversions are common in Perciformes. In fact, two-armed chromosomes have been found in approximately 30% of Carangidae species karyotyped to date (Chai et al. 2009). Furthermore, other kinds of chromosomal diversification have been identified for this family including Robertsonian translocations, transient in *Seriola* Cuvier, 1817 (Vitturi et al. 1986, Sola et al. 1997) or already established in *Selene setapinnis* (Mitchill, 1815) (Jacobina 2012).

**Figure 2.**
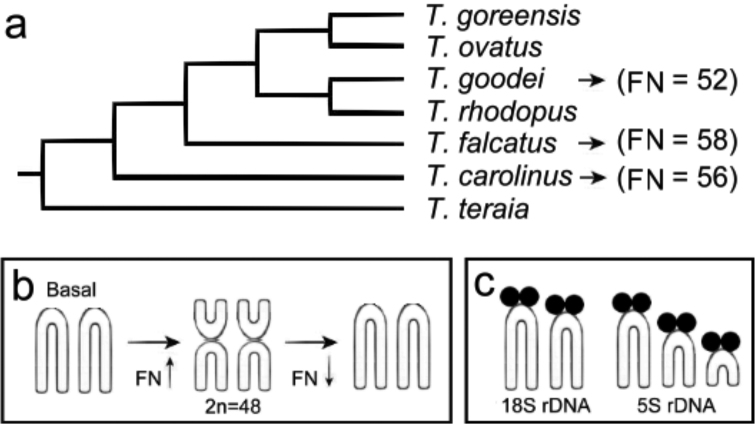
Phylogenetic tree from molecular data of some species of Trachinotini tribe (**a**), adapted from Reed et al. (2002). The molecular relationship is confronted with the chromosomal formula of the *Trachinotus* species analyzed. Schematic illustration shows the role of additional pericentric inversions leading to new acrocentric chromosomes and modification of the FN value (**b**), and the derived condition of multiple sites of 18S and 5S rDNAs in *Trachinotus goodei* (**c**).

Basing on morphological and molecular evidences, the genus *Trachinotus* is included in the tribe Trachinotini, which is considered one of the least diverse groups among carangids (Smith-Vaniz 1984, Gushiken 1988). Phylogenetic hypotheses based on mitochondrial sequences (Reed et al. 2002) suggest *Trachinotus carolinus* as the most basal species, followed by more derived *Trachinotus falcatus* and *Trachinotus goodei*, respectively.However, these phylogenetic relationships do not agree with the karyotypic characteristics presented by these species (Figure 2a).

Whereas the fully acrocentric karyotype with 2n=48 (FN=48) is considered basal for Perciformes, variations of this karyotypic formula can be interpreted as derived conditions. Thus, an increase in the number of two-armed chromosomes, as sequentially found in *Trachinotus carolinus* (eight two-armed chromosomes) and in *Trachinotus falcatus* (ten two-armed chromosomes), would be expected to represent derived cytogenetic characteristics. As such, *Trachinotus goodei*, showing only four two-armed chromosomes and, consequently, the largest number of acrocentric chromosomes, would be representing the species with the karyotype closer to the basal one.

Many closely related species of Perciformes show poorly varied or cryptic cytogenetic characteristics, hampering their application in phylogenetic inferences (Molina 2007, Motta-Neto et al. 2011a, b, c). Indeed, this is observed in the similar karyotype macrostructure or heterochromatic patterns, such as those found in *Trachinotus* species, where C-bands are inconspicuous and similarly located in the pericentromeric region of the chromosomes. A reduced amount of heterochromatin is also a common feature in other Perciformes, possibly resulting in lower karyotype evolution dynamics (Molina and Galetti 2004, Molina 2007). On the other hand, NORs were prominent characteristics, in lines with considerable karyotype variation between species. *Trachinotus carolinus* and *Trachinotus falcatus* displayed only one pair of chromosomes carrying ribosomal sites (Ag-NOR/18SrDNA/CMA^+^/DAPI^-^). This condition is considered basal and the most common for Carangidae (Caputo et al. 1996, Sola et al. 1997). As previously confirmed (Accioly et al. in press), the *Trachinotus goodei* population from Brazilian Northeastern coast exhibits a more derived condition, with two chromosomal pairs carrying ribosomal sites (pairs 5 and 11). Although multiple sites have not been identified in populations from the Southeastern coast (Rodrigues et al. 2007), the occurrence of more than one chromosome pair carrying NORs in *Trachinotus goodei* indicates some level of derivation in this species in relation to the others. Greater dynamic evolution of the ribosomal sites in this species is corroborated by the presence of three chromosomal pairs carrying 5S rDNA sequences (pairs 9, 12, 22), a condition not present in *Trachinotus carolinus* and *Trachinotus falcatus*, where these sites were mapped only in pair 9 (Figs 1, 2c). In addition, dual-color FISH showed no synteny between 18S and 5S rDNA sites in all the three species of *Trachinotus* analyzed here.

Simple ribosomal sites are considered an ancestral condition, most frequently found in carangids (Caputo et al. 1996, Sola et al. 1997), as well as among marine Perciformes (Galetti et al. 2000). Their location in distinct chromosomal pairs is an efficient cytotaxonomic marker of species and populations of *Trachinotus* (Accioly et al. in press). Indeed, Southeastern populations of *Trachinotus falcatus* and *Trachinotus goodei* are characterized by having simple Ag-NOR sites on the short arms of pair 18 and on the short arms of pair 3, respectively. The greater dynamic evolution of the 18S and 5S ribosomal sequences in *Trachinotus goodei* corroborates its more derived condition in relation to the other species (Figure 2), as suggested by molecular data (Reed et al. 2002). In turn, sharing of 5S rDNA sequences by a same chromosome pair, tentatively identified as no. 9, probably indicates homeologous chromosomes with similar syntenic content. The occurrence of three pairs carrying 5S rDNA sequences (pairs 9, 12 and 22) in *Trachinotus goodei* is uncommon among fish (Martins and Galetti 2000). The location of 5S and 18S rDNA sites in different chromosomes, and the functional divergence between 18S rDNA (transcribed by RNA polymerase I) and 5S rRNA genes (transcribed by RNA polymerase II) (Martins and Galetti 2000), supports the independent evolution of these multigene families due to specific selection pressures (Amarasinghe and Carlson 1998).

Variations in the number and location of NORs in some cases, are likely to be favored by a high and heterogeneous heterochromatic content, whereas the inverse seems to reduce the evolutionary dynamism of these regions (Molina 2007). Besides increasing the NORs’ dynamics, there are also indications that heterochromatin may act as hotspots for chromosomal rearrangements (Almeida-Toledo et al. 1996; Jacobina 2012). However, there is currently no information that the heterochromatin may be exerting some role in the evolutionary dynamics of the rDNA in *Trachinotus goodei*. Dispersion of these sequences in the karyotype may occur via transposition events by mobile elements in the carrier genome, with subsequent amplification and formation of new repetitive DNA sites (Eickbush and Eickbush 1995; Almeida-Toledo et al. 1996). Indeed, a surprising chromosome spreading of associated transposable elements and ribosomal DNA (Rex3/5S rDNA) was demonstrated to occur in the freshwater fish *Erythrinus erythrinus* (Bloch et Schneider, 1801) (Erythrinidae), increasing the number of such rDNA sequences from 2 to 22 between distinct populations (Cioffi et al. 2010). Growing knowledge on the organization of repetitive DNAs also indicates that their evolution may be mediated by unequal crossover, transposition mediated by RNA and gene conversion (Dover 1986, Martins et al. 2006). Thus, different events may be associated with the serial repetition of the 5S rDNA multigene family in the genome of *Trachinotus goodei*, characterizing its more derived condition in relation to the other species, *Trachinotus falcatus* and *Trachinotus carolinus*.

The existing set of cytogenetic data for Carangidae suggests karyotype evolution strongly mediated by pericentric inversion events. Based on the basal karyotype for Perciformes (2n=48 acrocentrics, FN=48), the increase of FN indicates a derived condition. Thus, if *Trachinotus goodei* is the most derived species in respect to *Trachinotus falcatus* and *Trachinotus carolinus*, as indicated by mitochondrial sequences (Reed et al. 2002), and supported by the apomorphic features of its karyotype (multiple 18S and 5S rDNA sites), a particular evolutionary pathway provided by pericentric inversions must be considered for this species. Thus, the smaller number of two-armed chromosomes in *Trachinotus goodei* may indicate additional rounds of pericentric inversions on two-armed chromosomes, increasing the number of acrocentric chromosomes in the karyotype and, consequently, decreasing the FN value (Fig. 2b). The same could be also considered for *Trachinotus carolinus*, considering its more basal position in the phylogeny proposed for *Trachinotus* (Fig. 2a).

Our understanding of the karyotype evolution of Carangidae (including rDNA) was improved by the present findings. Our data demonstrate that, in addition to structural changes by pericentric inversions, rDNA sequences also acted as an important evolutionary indicator in *Trachinotus* karyotype. In this sense, the combined mapping of 18S and 5S rDNA sequences proved to be useful to clarify the relationships in this fish group.
